# Influence of irregular shelterwood treatments on intensity and severity after a large wildfire in lodgepole pine stands: A case study from the interior British Columbia

**DOI:** 10.1371/journal.pone.0311940

**Published:** 2024-11-14

**Authors:** Mingrui Liu, Gregory Greene, Jodi Axelson, Nicholas Coops, Ignacio Barbeito, Dominik Roeser

**Affiliations:** 1 Faculty of Forestry, University of British Columbia, Vancouver, BC, Canada; 2 Department of Forest and Conservation Sciences, Faculty of Forestry, University of British Columbia, Vancouver, BC, Canada; 3 Office of the Chief Forester—Forest Science, Planning and Practices Branch, B.C. Ministry of Forests, Victoria, BC, Canada; Spanish Scientific Research Council, SPAIN

## Abstract

Climate change has significantly impacted the wildfire regimes in lodgepole pine forests, resulting in prolonged fire seasons and altered fire behaviour. In North America, fire patterns have shifted towards more frequent and severe wildfires after a century of fire suppression. In response, silviculture practices in fire-prone areas should aim to restore diverse forest structures that are resistant or resilient to wildfires. In Western Canada, where forestry is a key industry, interest in seeking silvicultural solutions for promoting forest resilience to wildfires has increased following the devastating wildfire seasons between 2017 and 2023. Irregular shelterwood, a silvicultural system with a relatively short history of implementation in British Columbia, has been deployed in ecologically sensitive areas to promote structural heterogeneity and meet management goals for biodiversity and wildlife values. Although the impacts of irregular shelterwood on wildlife habitat and abundance have been well studied, the interaction between wildfire and the stand structure created by irregular shelterwood remains poorly understood. To understand the effectiveness of the irregular shelterwood in building wildfire resilience, we present a study of a lodgepole pine stand that was treated with irregular shelterwood and partially burned in a wildfire in 2017. This study collected ground fuel, canopy fuel, and tree data from four stand types (irregular shelterwood treated-burnt, treated-unburnt, untreated-burnt, and untreated-unburnt) and analyzed the difference in char height and fire-induced mortality between burnt and unburnt conditions, with irregular shelterwood treatment being a variable. The results demonstrated reduced wildfire effect in the irregular shelterwood stand in this region of British Columbia. This observation was made at a stage where the openings have not been colonized by regeneration. This case study provides valuable insights into the effectiveness of irregular shelterwood in mitigating wildfire risk, and proposes a potential silviculture solution to promote forest resilience to wildfire.

## Introduction

Historically, the wildfire regimes of western North America were highly diverse, with a complex mix of severities and return intervals among different regions [1–4]. In the pre-settlement era, Indigenous communities in western US and Canada sustainably managed wildfires for centuries through silvicultural techniques such as controlled, selective burning, mimicking the effects of low- to moderate-severity disturbances [2, 5–8]. Mosaics of variable stand and fuel structures were created across the landscape as a consequence of small-scale burnings, and therefore limited the occurrence of stand-replacing wildfire events. However, two centuries of fire exclusion have altered forest structure and composition, and generated novel wildfire regimes [1, 3, 9–12], thereby compromising the capacity of forested ecosystems to resist or recover when fire occurs. For example, in western Canada, fire exclusion has extended the natural rotation age and increased the proportion of lodgepole pine *(Pinus contorta ssp*. *latifolia)* stands that are in late seral stages, thereby increasing the susceptibility of these forests to mountain pine beetle infestations [13]. The high prevalence of lodgepole pine stands with beetle infestations results in an increased accumulation of flammable materials post-outbreak, which in turn elevates wildfire hazard. Consequently, many western North American lodgepole pine forests have become denser and more susceptible to high-severity fires. These fuel alterations have transformed the wildfire regime to favor more frequent catastrophic fires that lead to significant economic, ecological, and human losses [2, 9, 14–16].

Canada’s western most province of British Columbia (BC) has witnessed escalating threats from wildfires. Between 2012 and 2023, where an average of 1,483 fires annually consumed approximately 407,373 hectares (ha) per year [17]. These wildfires have cost a total of $2.65 billion Canadian dollars in fire suppression effort over this period, a figure that excludes additional costs related to the evacuation of homes and communities [17]. Moreover, the 2023 fire season alone impacted an area of more than two million hectares, breaking previous records set in 2017, 2018 and 2021, and marking the worst wildfire season on record in the province [18]. Although not affected by the 2023 fires, the Quesnel Timber Supply Area (TSA), located near the center of BC, was significantly impacted in 2017, with fires burning through 22.5% of its timber harvesting land base. This wildfire damage resulted in a sharp decline in the allowable annual cut for Quesnel TSA, dropping from 2.6 million cubic metres to a midterm projection of 1.45 million cubic meters until 2075 [19, 20].

Silviculture started as a discipline aiming to optimize timber harvesting and economic return (REFERNCE). As the discipline has developed, it has integrated a better understanding of forest ecology; silviculture is now deployed in forest management to help forests adapt to climate change by creating compositional and structural complexity throughout the landscape [21–29]. Recent research examining silviculture treatments, especially thinning combined with pruning or prescribed burning, has underscored their effectiveness at decreasing fire mortality and moderating fire behavior in various forest types [30–37]. However, there is a limited body of research investigating fire effects in partial cutting systems using shelterwood or selection cuts.

In BC, clearcutting with reserves has been the dominant silvicultural system on public lands since 1987 [38]. Among the few partial cutting practices used, irregular shelterwood is one of the few legally sanctioned silvicultural systems for harvesting in wildlife habitat areas (WHA), and is the designated silvicultural system for regions in central BC to ensure terrestrial lichen supply for mountain caribou, and has wide implementation in the Quesnel TSA [39, 40]. The irregular shelterwood is a more complex variant of other shelterwood systems (e.g., uniform, strip), distinguishing itself by prolonged regeneration periods and spatial variability for enhancing structural heterogeneity, biodiversity and regeneration [41, 42]. This silvicultural system creates irregular opening patterns in the residual stand. The dead fine and coarse woody debris accumulated during harvesting could generate a potential trade-off between wildlife conservation and fire hazard [43–45]. Previous research on the use of irregular shelterwoods has underscored its positive effects on BC’s caribou habitat quality, bird species conservation, understory vegetation, and tree species richness and abundance [46–49]. However, this silvicultural system’s influence on wildfire dynamics remains underexplored, especially given the potential role of structural heterogeneity in moderating wildfire behaviour [50–52]. Given escalating wildfire extent and severity since the 2000s, understanding both the response and effects of wildfire to irregular shelterwood systems is crucial.

The objective of this study is to evaluate how an irregular shelterwood treated stand responded to a 2017 wildfire in the central interior of BC. We hypothesize that the wildfire exhibited reduced fire intensity in response to the treatment, which in turn reduced severity compared to the wildfire response and effects in an untreated stand. To test this hypothesis, we first investigated differences in the stand structure and composition of a treated and untreated stand. We then investigated whether there were any significant differences in char height and live tree survival between the treated and untreated stands to evaluate differences in fire behaviour and effects. The results from this case study will help design silvicultural approaches that enhance the resilience of forested stands to wildfire.

## Materials and methods

### Study site

**Fig 1:**
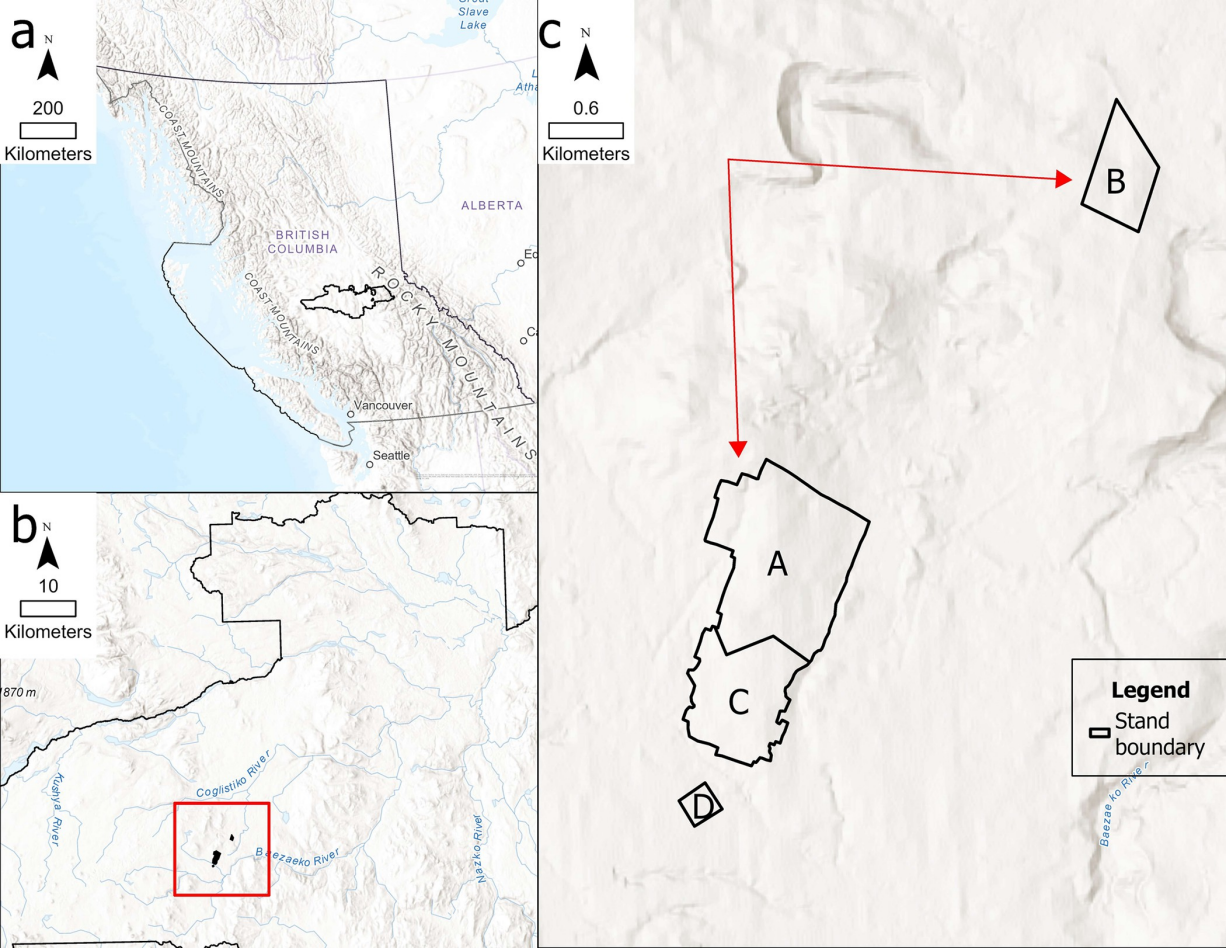
(a) Province boundary of BC and Quesnel TSA (b) Location of study site in Quesnel TSA (c) Stand boundaries of study site. Red arrows in Fig 1 (c) show the spread direction of the wildfire in 2017. Stand type: A: treated-burnt; B: untreated-burnt; C: treated-unburnt; D: untreated-unburnt.

The study site is located in a lodgepole pine forest 100 km west of the city of Quesnel, situated in the Quesnel Timber Supply Area (TSA) of central BC (Fig 1A and 1B). Central BC includes the Chilcotin and Cariboo plateaus, part of Nechako Plateau, and the Bulkley, Tahtsa and Chilcotin Ranges. The Chilcotin Plateau, where the study site lies, features a flat or rolling landscape lying between 1200 to 1500m elevation with many streams, wetlands and lakes [53].

This region is categorized within the Montane Spruce (MS) biogeoclimatic (BEC) zone, and the study area is specifically located in the very dry and very cold (xv) subzone, with a mean annual temperature of 0.3 degrees Celsius, and 537mm annual precipitation [54, 55]. The MSxv zone is characterized by forests dominated by mature and near mature lodgepole pine (85%) with minor amounts of interior hybrid spruce (15%)*(Picea engelmannii x glauca)* [56]. Natural disturbance type (NDT) 3 is the dominant disturbance regime in MSxv zone, being defined as frequent stand-initiating events occurring every 100 to 150 years on average [57]. Historically, the forests in this zone experienced frequent mixed-severity wildfires that ranged in sizes, and the last wildfire burnt in the 2017 and 2018 fire season [58]. A mountain pine beetle outbreak from 1990 to 2010 killed most of the mature lodgepole pine and large diameter immature pines, resulting in a widespread live sub-canopy layer of immature pine and spruce.

Within the 250 ha study area, the forest is comprised of nearly even-aged canopy layer of almost pure lodgepole pine, with minor components of hybrid spruce (10%) that are 120 to 150 years old, according to the provincial vegetation resource inventory (VRI) data [59]. This stand is classified as old growth forest, according to the definition provided by the British Columbia government, which typically considers dry interior forests to be old when they reach 140 years of age [60]. The lodgepole pine forests in this region have an average of 50–60% mortality as a result of the mountain pine beetle endemic in the early 2000s [59]. This forest includes many terrestrial lichen sites, which are important food sources for the northern mountain caribou herds living there [61]. Under the regulation of BC provincial general wildlife measures [39], an irregular shelterwood silvicultural system aiming for 50% canopy openings was implemented in 2012 to fulfill the harvesting requirement on terrestrial lichen sites. Small patches of 0.15 ha (30m x 50m) were harvested, while retention patches of the same size were left surrounding each opening to provide canopy shelter, giving the irregular shelterwood treatment area a checkerboard appearance (Fig 1C).

Satellite images taken in the summer of 2017 showed that a wildfire originating from the northwest approached the study site and divided into two distinct branches, as illustrated by the red arrows in Fig 1C. One branch progressed southward, while the other advanced eastward, converging on the northern half of the irregular shelterwood treatment and an adjacent untreated stand in the same day. Due to the lack of records, it was difficult to determine the exact ignition spot of the fire in polygon A. Therefore, we used historical aerial photos to infer the ignition spot; a Landsat photo dated August 4, 2017, reveals the imminent spread of the fire into polygon A from the north-west corner. Based on this fire spread pattern and this observation, the ignition spot was inferred to be on the north-west corner of polygon A (Fig 1C). This wildfire was naturally extinguished by precipitation on August 12^th^, approximately in the middle of polygon A and C. The boundary of the burnt and unburnt stands is visible in Fig 1C. Lodgepole pine is not conventionally considered fire tolerant due to its thin bark [62], however, the irregular shelterwood lodgepole pine stand displayed signs of reduced fire severity compared to an untreated stand in the satellite image (Fig 1C). This fire event presented a unique opportunity to examine the response of this silvicultural system to wildfire in a lodgepole pine leading forest.

### Sampling design

The primary objective was to compare fire effects between treated-burnt (Stand A) and untreated-burnt (Stand B) areas. To understand these effects, we needed a baseline–fuel conditions in similar stands before the fire. Historical VRI data [59] from 2016, one year prior to the fire, was used to find pairwise stands of the similar attributes to the treated-burnt and untreated-burnt stand, respectively, to reflect their pre-fire fuel conditions for modelling purposes. BC VRI data is an open-access inventory that maps the stand attributes across the whole province and is updated every year. It is done at the stand level based on a mixture of measurements, so its resolution and accuracy are not ideal for this purpose. However, it sets a reasonable baseline and is commonly used to approximate the stand attributes when field survey data is not available.

The treated-burnt stand displayed comparative stand attributes with the treated-unburnt stand, as did the untreated-burnt stand with the untreated-unburnt stand (Table 1). Using a space-for-time substitution, we assume (1) the two unburnt stands represent the pre-fire fuel conditions of their burnt counterparts; and (2) the pre-fire fuel configurations are analogous between treated-unburnt-retention and untreated-unburnt stands. This similarity supported our goal for Stands C and D to accurately represent the pre-fire conditions of Stands A and B, respectively.

**Table 1:** Stand attributes before the fire event, derived from 2016 BC vegetation resources inventory data [58].

Stand Type	Code	Species composition (%)		Live tree density (stems ha^-1^)	Dead tree density (stems ha^-1^)	Age (yr)
		Pli	Sx			
Treated-Burnt	A	80	20	270	200	128
Untreated-Burnt	B	85	15	459	500	143
Treated-Unburnt	C	80	20	270	200	128
Untreated-Unburnt	D	100	0	500	600	143

Pli, lodgepole pine; Sx, Hybrid spruce (Picea engelmannii x glauca)

### Assumption and reasonings

The unpredictable nature of wildfires, including their timing, location, and characteristics, poses significant challenges to establishing an ideal pre-fire experimental design. In light of these challenges, the best approach to assessing fire effects is to identify geographically adjacent treated and untreated stands that share similar ecology and terrain features [30, 63]. Adhering to this criterion, the unburnt stands (treated-unburnt and untreated-unburnt) are assumed to emulate the pre-fire conditions of their burnt counterparts.

Unexpectedly, the treated-unburnt-retention stand had slightly lower live and dead tree densities compared to the untreated-unburnt stand. This variation can be attributed to several observed field data factors. Sporadic natural openings were observed throughout the untreated-unburnt stand and are mirrored in the standard deviation for both its live and dead tree densities. This is a common result from canopy opening at the old-growth stage [64–66] and was the same in our study stand, which is classified as an old-growth forest. Furthermore, the presence of windthrow trees at the edges of each retention patch in the treated-unburnt-retention stand resulted in reduced densities for both live and dead trees. Such windthrow impact at the edges has been similarly recorded in other studies on different group retention treatments [67, 68]. Finally, differences observed between stand types may also be a result of the relatively small sample size used in this study.

In this study, live fuel loading, including shrubs and moss on the surface or along the tree bark, was assumed to have minimal effect on the wildfire behaviour and was excluded from sampling. This assumption is based on field observations indicating a very light live fuel presence in each plot, with conditions being highly similar between the treated-unburnt and untreated-unburnt stands. However, this assumption comes with limitations. The moisture content dynamics of the live fuel loading can impact the fire behaviour. High moisture content can decelerate the rate of spread of wildfire whereas moisture content below 120% in prolonger fire seasons can convert live fuel loadings into dead herbaceous fuel loadings [69, 70]. Despite the low presence of live fuel loadings in our plots, the exclusion of them from sampling might impact the fire intensity and thus lead to errors associated with measured fire intensity indicators.

### Field sampling

**Fig 2:**
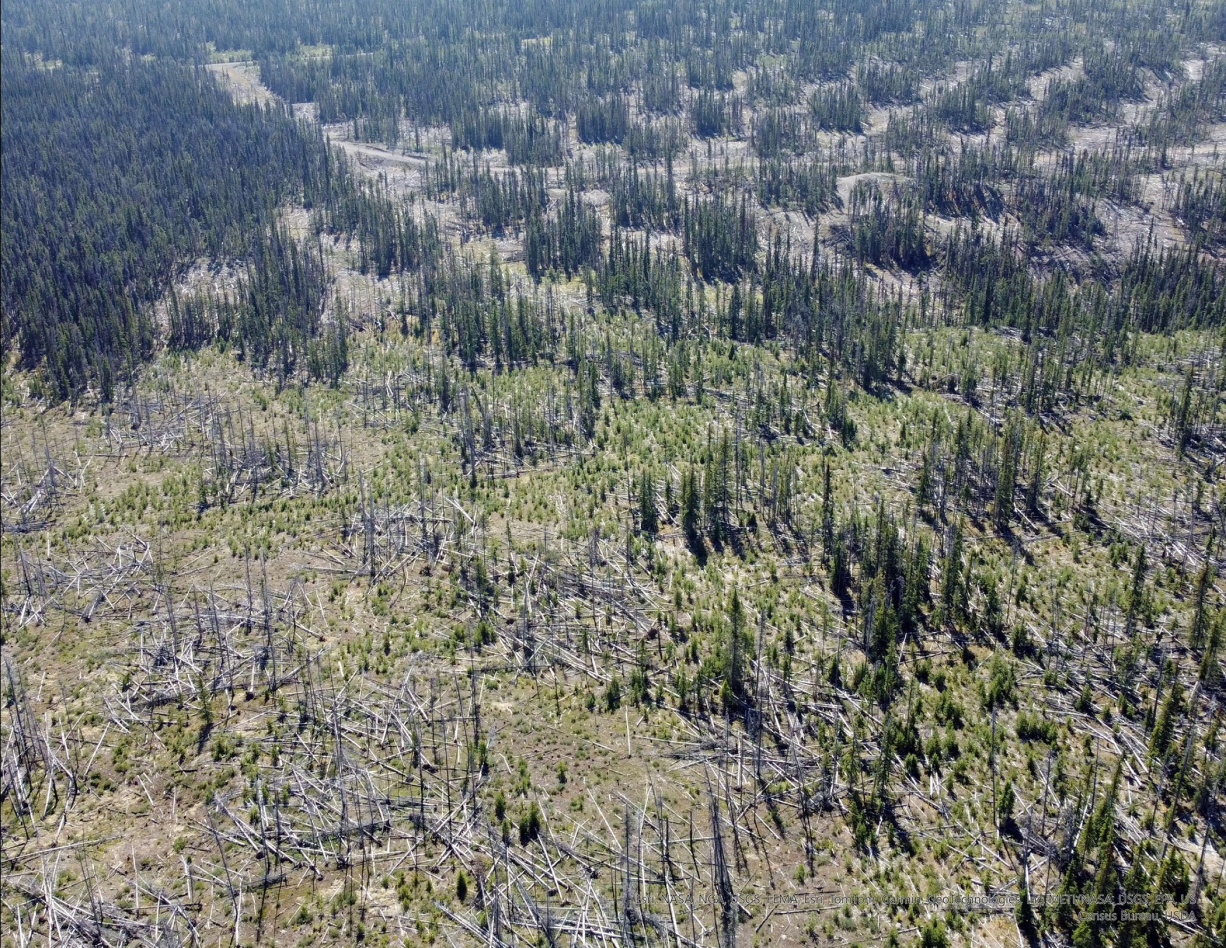
Aerial photo of the irregular shelterwood stand. Bottom-left corner shows part of the treated-burnt stand with retentions and openings. Bottom right shows treated-unburnt stand in the same way. Top right shows a recently harvested irregular shelterwood stand to give readers a better idea of what this system looks like. Top left shows a small patch of unburnt stand.

This study involved field research. Field data collection was carried out in the crown land in the province of British Columbia. No field permit was needed or issued. BC Ministry of Forests granted permission for conducting the field research. Field sampling was carried out in the study area in the winter of 2022 and spring of 2023. In total, there were six land cover types in this study: treated-burnt-retention, treated-burnt-opening, treated-unburnt-retention, treated-unburnt-opening, untreated-burnt and untreated-unburnt (Fig 2). Key assumptions are: (1) the two unburnt stands represent the pre-fire conditions of their burnt counterparts; (2) the pre-fire fuel conditions are analogous between treated-unburnt-retention and untreated-unburnt stands; and (3) the weather conditions are uniform for the four stands during the fire event due to their spatial proximity. The main steps of field sampling are summarized in Table 2. Each step is explained in detail as follows.

**Table 2:** Summary table of main sampling steps.

Field sampling steps	Variables measured
Fixed radius plot measurement	DBH, Species, Height(m), Char height(m)
Ground fuel sampling	Ground fuel loading by size classes (kg m^-2^)
Live tree tally along skidding trails	Number of post fire live tree in each retention patch
Char height measurement along skidding trails	Average char height from the 5 closest tree to patch center

**Fig 3:**
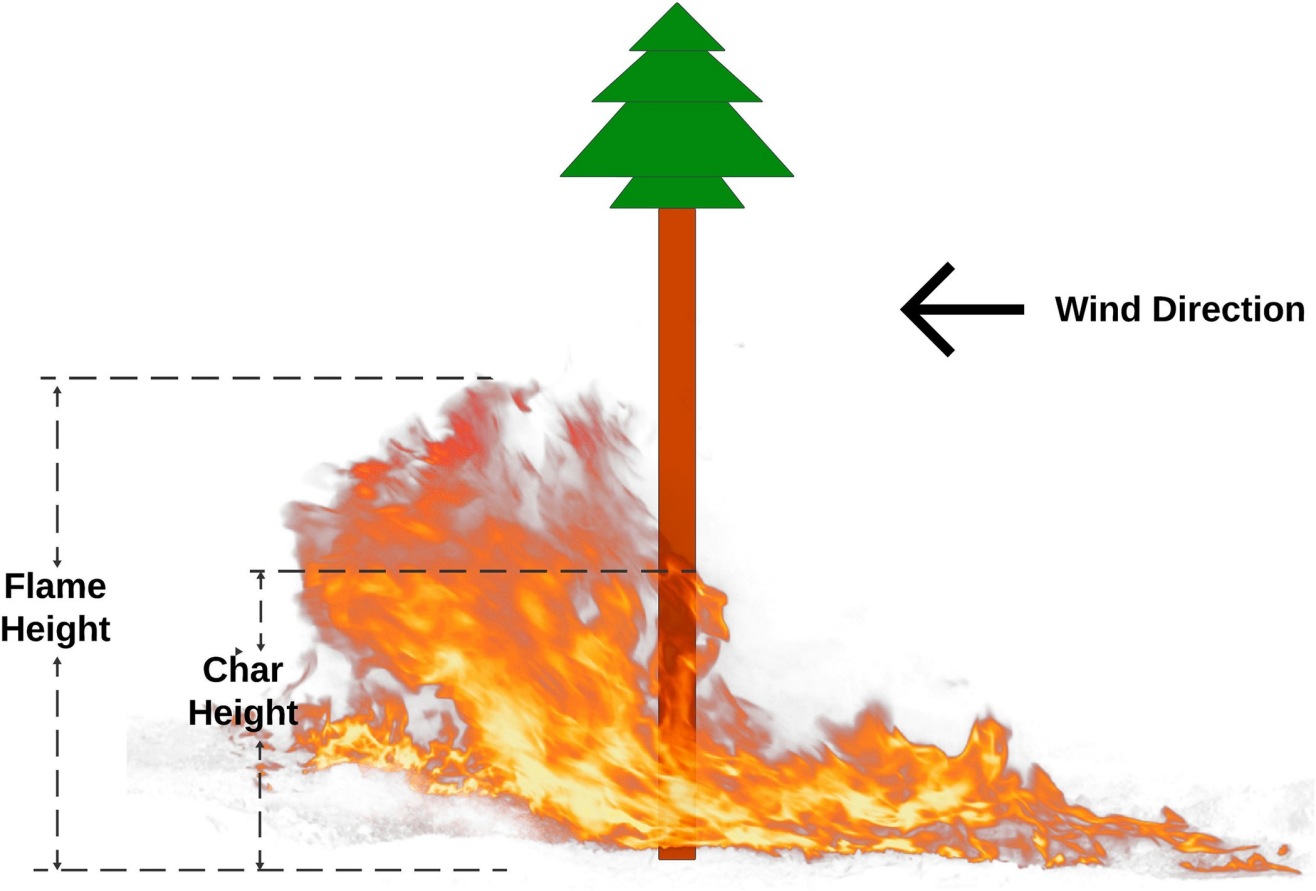
Definition of char height and flame height.

Six plots were randomly established within each land cover type. In total, 36 fixed radius plots of 11.28m radius were established to measure overstory trees (DBH ≥ 12.5cm) in the plot. A nested plot of 5.64m radius was established on the same plot center to measure all small trees (DBH ≥ 5cm but < 12.5cm). Tree measurements included: status (live or dead), species, diameter-at-breast-height (DBH, cm), and tree height (m). To evaluate fire effects, standard fire severity metrics [71] were measured on plot trees within treated-burnt and untreated-burnt stands. Bole char height, indicative of flame height (Fig 3), was measured to the nearest 0.1 centimeter from each tree within each 11.28m plot. Beetle attacked standing dead trees can result in significantly higher flame length and torching potential than unaffected healthy trees [72, 73]. Considering that each plot has a mixture of beetle-attacked dead and healthy trees, averaging the measured char height from both classes will result in significant variance. During field sampling, beetle-attacked dead trees in burnt plots were easily distinguishable due to their advanced decay, which included sloughing bark, reduced large branches, and visible sapwood and root rot. These trees had visible beetle holes, were more flammable than live trees, and often had fire char extending into the xylem. Due to their potential to produce higher flames, including them in char height sampling could bias the results, so they were excluded. In the cases where part of the plot extended past the intended cover class, the mirage method [74] was adopted to determine the corresponding inclusive zones [75]. The boundary of the exceeding part was visually flipped back into the plot and marked. Every tree entry within this inclusive zone was duplicated.

Within each plot, one 30m long transect bisected by plot centre was established on a random azimuth. Sampling was done along the direction of the azimuth using the line intercept method [76]. Fine Woody Debris (FWD; diameter < 1cm) was classified into two size classes (diameters 0–0.5 cm and 0.5–1 cm), and tallied from 0-5m and 0-10m along the transect, respectively. Medium Woody Debris (MWD; diameter ≥ 1cm– 7cm) and Course Woody Debris (CWD; diameter ≥ 7cm) were divided into four classes (1–3 cm, 3–5 cm, 5–7 cm, ≥ 7 cm), and sampled from 0-15m, 0-20m, 0-25m, and 0-30m along the transect, respectively. All MWD and CWD diameters were measured with a caliper to increase the accuracy of the fuel load estimations [77]. At 7.5 and 22.5 meter on the transect, surface layers were removed to the mineral soil and litter and duff layers were visually identified and measured to the nearest millimeter with a ruler. The reflection method was followed [78], in which the section of the transect that went out of the intended cover class was flipped back into the block, overlaying on the remainder of the transect. All surface fuels were sampled twice in the overlapped section of the transect.

In addition to the plot measurements above, in the treated-burnt stand, the total count of live trees was tallied from retention patches on both sides of the three primary skidding trails. The objective was to capture potential changes in fire effects as it spreads through the irregular shelterwood-treated stand. Likewise, char height was measured from the five nearest trees by the patch center within every 3^rd^ retention patch along either side of the three main skidding trails.

### Field data processing

All sampled trees were classified into four layers based on the measured DBH (Layer 1: DBH ≥ 12.5cm, Layer 2: 12.5 > DBH ≥ 7.5cm, Layer 3: 7.5 > DBH ≥ 5cm, Layer 4: DBH < 5cm). Mean and Standard Deviation (SD) for density, tree height, and quadratic mean diameter (QMD) were calculated separately for the live and dead trees in these four layers in each of the four cover types.

Woody debris fuel loading was calculated for fuels < 7cm diameter (FWD, MWD) following the procedures outlined by Van Wagner [79]. Each size class was first assigned a slope adjustment factor to convert the transect length to horizontal:

$$Co_{slope}=\sqrt{1+{slope}^{2}}$$

$$l_{cor}(m)=l/Co_{slope}$$

where Co_slope_ is the slope adjustment factor, slope (%) is the plot slope measured at the plot center, l_cor_ is the horizontal length of the transect after adjustment, and l is the transect length (m) which equals 5m and 10m for the 0–0.5cm, 0.5-1cm fuel size classes, respectively. The loadings were then calculated using the following equations:

$${Volume}_{FWD}=10000*n*\frac{0.1234}{l_{cor}}*{\left(\frac{qmd}{100}\right)}^{2}$$

$${Loading}_{FWD}=Co_{slope}*g*{Volume}_{FWD}$$

where *Volume* is the calculated volume (m^3^) for the fuel size class under calculation, *n* is the total number of tallies for one of the two FWD fuel size classes under calculation, *l*_*cor*_ is the horizontal transect length (m), and *qmd* is the quadratic mean diameter of each fuel size class, which was calculated following Van Wagner’s (1982) theoretical power law distribution approach [79]. *Loading* is the converted loading per unit area (kg/m^2^) for the fine fuel size class under calculation, and *g* is specific gravity, which was applied as a weighted average value of 0.468 for both fuel size classes. This value reflects a study area-wide species composition of 80% lodgepole pine (g = 0.49) and 20% hybrid spruce (g = 0.38) [79–81]. FWD fuel loading was calculated for both size classes in this manner.

Woody debris fuel loads were calculated for all fuels ≥ 1 cm diameter (i.e., all MWD & CWD fuel size classes) as follows. Each piece was first converted to a fuel load using the following equation:

$$Volume=10000*\frac{0.1234}{l_{cor}}*{\left(\frac{diam}{100}\right)}^{2}$$

$$Loading={Co}_{slope}*g*Volume$$

where *Volume* is the calculated volume (m^3^) for each piece, *l*_*cor*_ is the horizontal transect length (m), and *diam* is the measured diameter for each piece. *Loading*_*MWD*_ is the calculated loading per unit area (kg/m^2^) for each MWD and CWD piece.

Fuel loadings for the United States time-lag fuel size classes (1-hr, 10-hr, 100-hr, 1000-hr, 10000-hr, greater than 10000-hr) were calculated from the FWD, MWD, and CWD fuel loading values as follows:

$${Loading}_{1-hr}={Loading}_{{FWD}_{0.5cm}}$$

$${Loading}_{10-hr}={Loading}_{{FWD}_{1cm}}+\sum_{i=0.1}^{2.539}{Loading}_{i}$$

$${Loading}_{100-hr}=\sum_{i=2.54}^{7.619}{Loading}_{i}$$

$${Loading}_{1000-hr}=\sum_{i=7.62}^{22.859}{Loading}_{i}$$

$${Loading}_{10000-hr}=\sum_{i=22.86}^{50.799}{Loading}_{i}$$

$${Loading}_{>10000-hr}=\sum_{i=50.80}^{\infty }{Loading}_{i}$$

where *Loading*_*1-hr*_, *Loading*_*10-hr*_, *Loading*_*100-hr*_, *Loading*_*1000-hr*_, *Loading*_*10000-hr*_, *Loading*_*>10000-hr*_ are the 1-hr (0–0.635 cm), 10-hr (0.635–2.54 cm), 100-hr (2.54–7.62 cm), 1000-hr (7.62–22.86 cm), 10000-hr (22.86–50.80 cm), and greater than 10000-hr (≥ 50.8 cm) fuel loading values (kg/m^2^), respectively. *Loading*_*FWD0*.*5cm*_ is the fuel load (kg/m^2^) for FWD fuels 0–0.5 cm diameter, *Loading*_*FWD1cm*_ is the fuel load for FWD fuels 0.5–1 cm diameter, and the sigma functions represent the sum of fuel loads for each piece of MWD and CWD fuel with diameter (*i*) that is within the diameter ranges listed. Due to the tallying of FWD fuels, the final fuel loading values assigned to *Loading*_*1-hr*_ and *Loading*_*10-hr*_ are not exactly equivalent, as there is a 0.135 cm difference in diameter ranges between the two size class systems. While this would lead to the slightest underestimation of *Loading*_*1-hr*_ and overestimation of *Loading*_*10-hr*_, the differences are assumed to be negligible.

Statistical tests were applied to assess the difference of mean woody fuel loading under each fuel size class in treated-unburnt-retention and untreated-unburnt stand. The Shapiro-Wilk test and Levene’s test were applied to all but the 100-hr time-lag fuel size class to test dataset normality and homogeneity of variances. Based on the results, we conducted different statistical tests for each time-lag fuel size class. Specifically, we performed standard t-tests for 1-hr, 10-hr, 1000-hr, and 10000-hr classes where the assumptions of normality and homogeneity of variances were met. For the greater than 10000-hr class, which violated the homogeneity of variances assumption, we applied Welch’s t-test because it is specifically designed to compare the means of two groups without requiring the assumption of equal variances. The 100-hr fuel size class was excluded from statistical testing due to its unique distribution, with all values in untreated-unburnt stand being zero. Such a distribution violates assumptions of normality and/or variance homogeneity required for parametric or non-parametric tests and limit their usefulness. Instead, the 100hr fuel size class was only visually assessed.

The measured char height was divided into two treatment types–those from treated stand and from untreated stand. All char heights over 1m were classified into 1m intervals from 1-2m to 13-14m. Char heights below 1m were divided into two intervals (0–0.5 m, 0.5–1 m) to capture differences in low intensity surface fires. The total counts of char height falling in each class were plotted to show the char height distribution difference between the treated and untreated stand.

### Distance effect of fire spread in treated stand

To predict post-fire live tree density within the retention patch, the distance from the ignition spot (i.e., the point of entry of the fire into the stand) to the center of the retention patch was used as the predictor variable. Given the discrete and count-based nature of the predictor variable, we decided to fit negative binomial model, which is particularly suited for count data characterized by overdispersion. The model was fitted using the ‘*glm*.*nb’* package in R (ver.4.2.1) [82, 83]. A deviance residual plot was visually assessed to ensure model’s goodness-of-fit. The significance of the predictor variable in forecasting the response variable was ascertained by interpreting the t-statistic (α = 0.05). Likewise, char height was measured from the five nearest trees by the patch center within every 3^rd^ retention patch along either side of the three main skidding trails. A linear regression model of char height was fitted using distance from ignition spot as predictor, using the *‘lm’* function in R to test the significance of the distance effect on reducing char height in this stand structure. The linear relationship, normality and homoscedasticity were visually assessed in the diagnostic plots to ensure model assumptions were met. There is assumed to be no multicollinearity or auto-correlation between average char height and distance from ignition spot.

## Results

### Stand attributes in four treatment types

**Fig 4:**
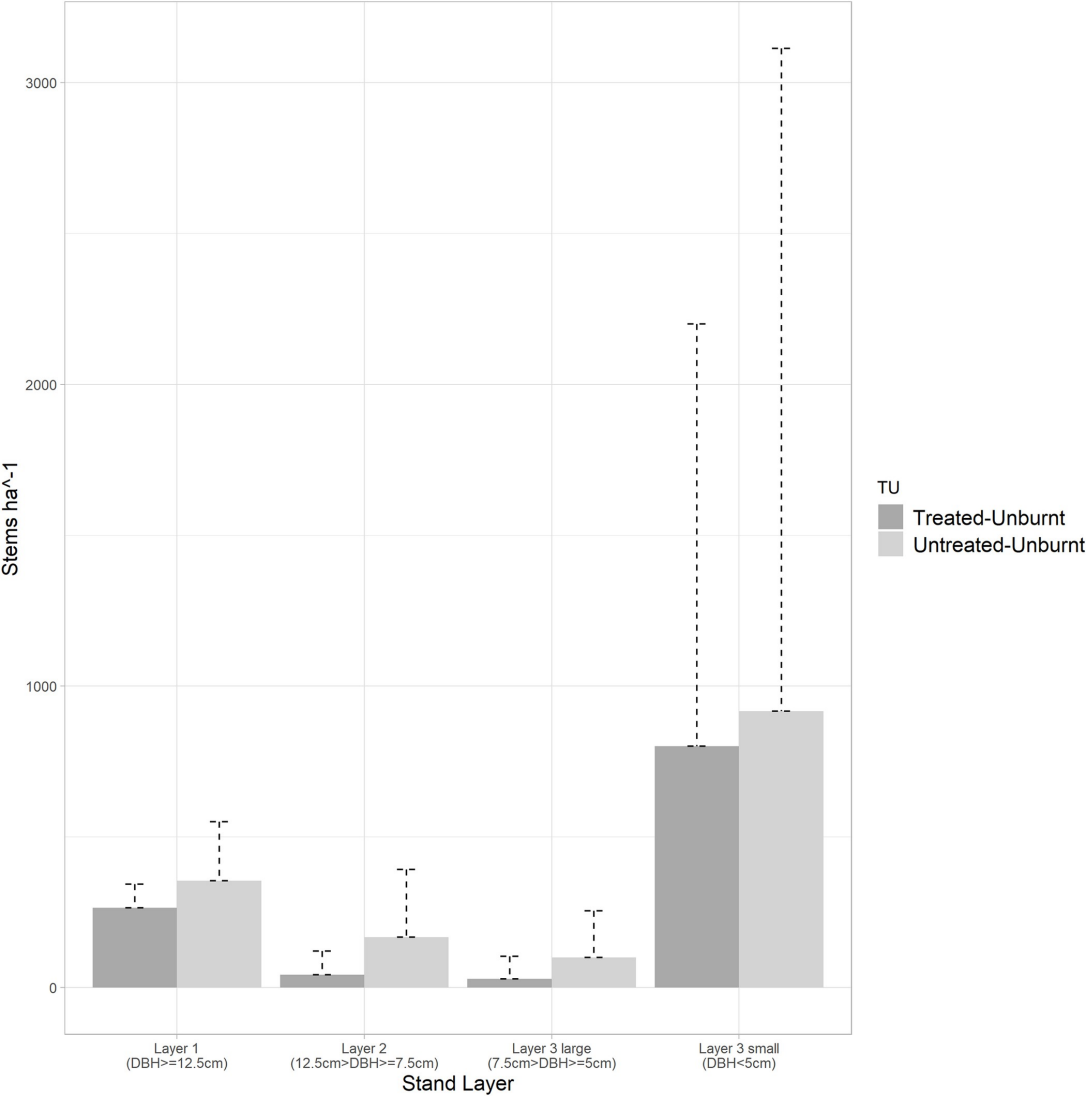
Live tree density in treated-unburnt-retention vs untreated unburnt stand.

Compared to the untreated-unburnt stand, the treated-unburnt-retention stand showed a slightly reduced density for live trees (264 stems ha^-1^ versus 354 stems ha^-1^) and dead trees (289 stems ha^-1^ versus 443 stems ha^-1^), particularly in layer 1, which comprises 88.5% of the overstory canopy (Table 3). The difference for live and dead tree density is greater in layer 2, accounting for 27% of the overstory canopy cover, in comparison to that of 12% in the treated-unburnt-retention stand, although accompanied by substantial variance. Regarding regeneration in the layer 3 class, both unburnt stands presented comparable counts of live saplings (800 vs 917 stems ha^-1^) despite significant variance, with the absence of dead saplings in both stands (Fig 4).

**Table 3:** Summary statistics of the four study stands.

	unburnt						Burnt					
	treated-retention			untreated			treated-retention			untreated		
Layer 1 (DBH ≥ 12.5cm)	mean	sd	se(n = 6)	mean	sd	se(n = 6)	mean	Sd	se(n = 6)	mean	sd	se(n = 6)
Live tree density (stems/ ha^-1^)	264(132)*	78.87	29.80949	354	195.20	79.69124	25(13)*	38.73	15.81139	0	-	0
Dead tree density (stems/ ha^-1^)	289(145)*	118.02	44.60713	433	218.90	89.36504	242(121)*	174.40	71.20003	350	109.54	44.72136
Live tree height (m)	13.75	1.83	0.693155	13.64	0.54	0.218686	12.85	2.25	1.299038	-	-	-
Dead tree height (m)	13.84	1.37	0.517446	14.49	1.28	0.523002	13.78	1.63	0.665759	15.33	3.91	1.59691
Live tree QMD (cm)	19.25	1.31	0.49387	18.38	3.91	1.597024	17.28	3.41	1.966661	-	-	-
Dead tree QMD (cm)	21.21	1.63	0.614939	21.52	3.53	1.442478	18.83	3.34	1.361914	18.10	2.43	0.990737
**Layer 2 (12.5cm > DBH ≥ 7.5cm)**												
Live tree density (stems ha^-1^)	43(21)*	78.68	29.73809	167	225.09	91.89366	0(0)*	-	0	0	-	0
Dead tree density (stems ha^-1^)	29(14)*	48.80	18.44278	133	242.21	98.88265	200(100)*	189.74	77.45967	233	250.33	102.1981
Live tree height (m)	7.20	0.14	0.1	7.38	3.29	1.901459	-	-	-	-	-	-
Dead tree height (m)	10.15	6.58	4.65	9.29	0.27	0.191667	7.58	2.34	1.168215	12.20	1.15	0.512791
Live tree QMD (cm)	10.25	1.06	0.749999	9.99	1.09	0.629126	-	-	-	-	-	-
Dead tree QMD (cm)	9.75	0.35	0.25	10.51	0.37	0.261569	9.75	0.94	0.469564	11.00	0.64	0.287232
**Layer 3 large (7.5cm > DBH ≥ 5cm)**												
Live tree density (stems ha^-1^)	29(14)*	75.59	28.57143	100	154.92	63.24555	0(0)*	0.00	0	0	0	0
Dead tree density (stems ha^-1^)	0(0)*	-	0	50	83.67	34.1565	17(8)*	40.82	16.66667	17	40.82	16.66667
Live tree height (m)	3.80	-	-	5.93	3.20	1.846017	-	-	-	-	-	-
Dead tree height (m)	-	-	-	4.10	1.56	1.1	4.80	-	-	2.60	-	-
Live tree QMD (cm)	5.50	-	-	6.59	0.37	0.216431	-	-	-	-	-	-
Dead tree QMD (cm)	-	-	-	6.05	0.35	0.25	7.10	-	-	5.10	-	-
**Layer 3 small (DBH < 5cm)**												
Live tree density (stems ha^-1^)	800(400)*	1400.00	700	917	2196.74	896.8154	0(0)*	-	-	0	-	-
Dead tree density (stems ha^-1^)	0(0)*	-	0	0	-	0	0(0)*	-	-	0	-	-
Live tree height (m)	2.30	0.54	0.26927	1.85	0.35	0.25	-	-	-	-	-	-
Dead tree height (m)	-	-	-	-	-	-	-	-	-	-	-	-
Live tree QMD (cm)	4.20	2.59	1.294216	2.12	1.87	1.324012	-	-	-	-	-	-
Dead tree QMD (cm)	-	-	-	-	-	-	-	-	-	-	-	-

*: The first value represents the stand density measured in treated-retention patches. The second value in brackets indicates the stand density for the entire treated stand, calculated by halving the density of treated-retention patches, in alignment with the treatment objective of achieving 50% canopy opening. sd remains the same as there was no tree in treated-opening patches.

-: ’-’ in mean columns means that no observation was available for that particular class. ’-’ is also used in sd columns where there is no observation or only one observation where sd is inapplicable.

Tree height and QMD for both live and dead trees within the two burnt stands presented similar values with small standard deviations (Table 3). A noteworthy observation was the complete absence of live trees in the untreated-burnt-retention stand, in contrast to the number of post-fire live trees (25 stems ha^-1^, SD = 38.73) observed within layer 1 of the treated-burnt-retention stand. Despite noticeable variances, the density of dead trees across all four layers exhibited consistency between the two burnt stands.

### Surface fuel loading in treated-unburnt vs untreated-unburnt

**Fig 5:**
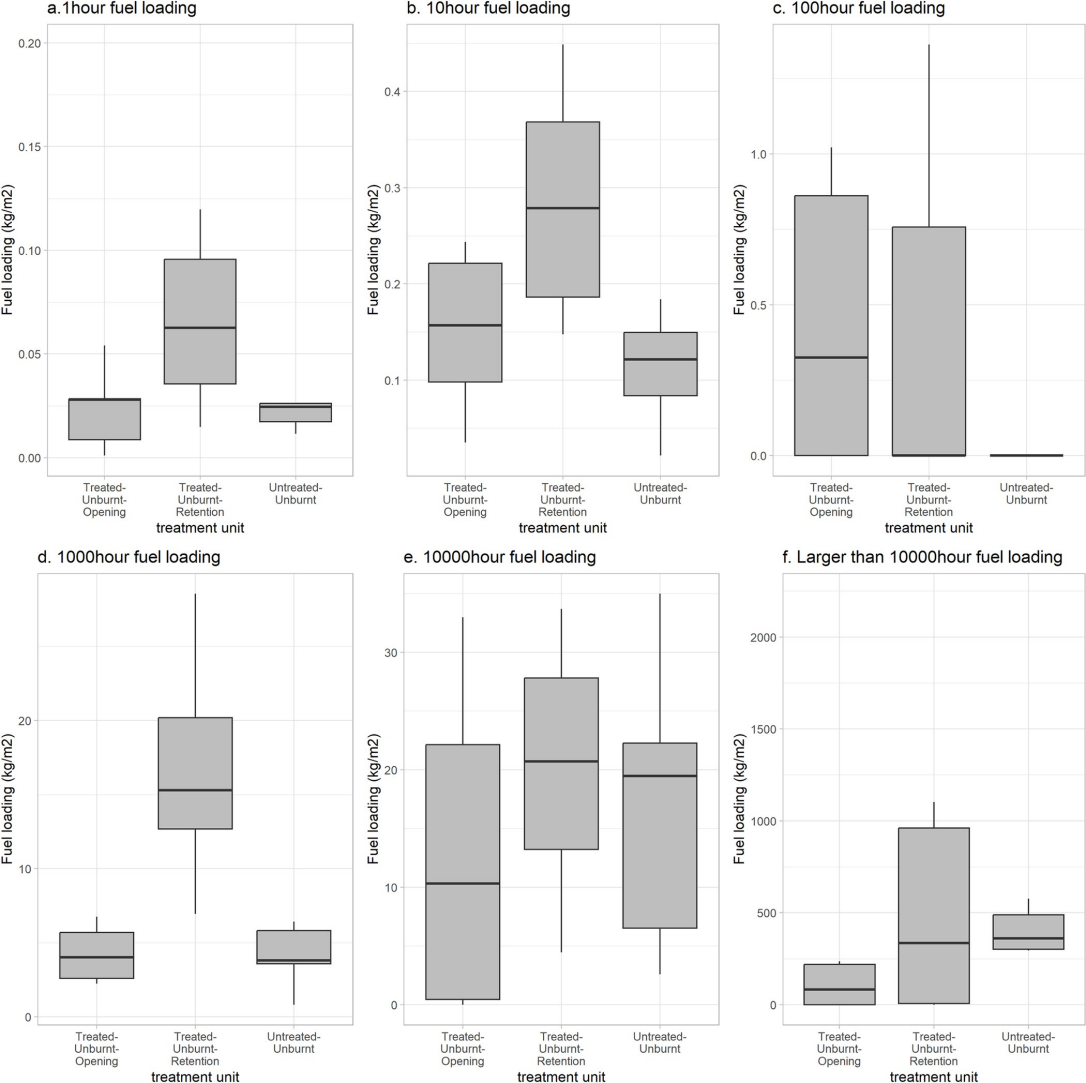
Ground fuel loadings by fuel size class (hr) in treated-unburnt stand vs untreated-unburnt stand.

Fuel loading was notably higher in 10-hr (p = 0.008) and 1000-hr (p = 0.003) time-lag fuel size class in the treated-unburnt-retention stand compared to the untreated-unburnt stand (Fig 5B and 5D). Fuel loading in the 1-hr time-lag fuel size class also appears to be higher in the treated-unburnt-retention stand but was not statistically significant (p = 0.08) (Fig 5A). Fuel loading derived from large, downed wood, characterized by the 10000-hr (p = 0.63) and greater than 10000-hr (p = 0.55) classes, was found to be similar between the treated-unburnt-retention and untreated-unburnt stand (Fig 5E and 5F). Fuel loading in 100-hr size class yielded a comparable mean of 0 (p value not applicable), with a large variance in the treated-unburnt-retention stand, indicating some scattered presence of fuel in this size class.

### Fire behaviour at plot level

#### Bole char height

**Fig 6:**
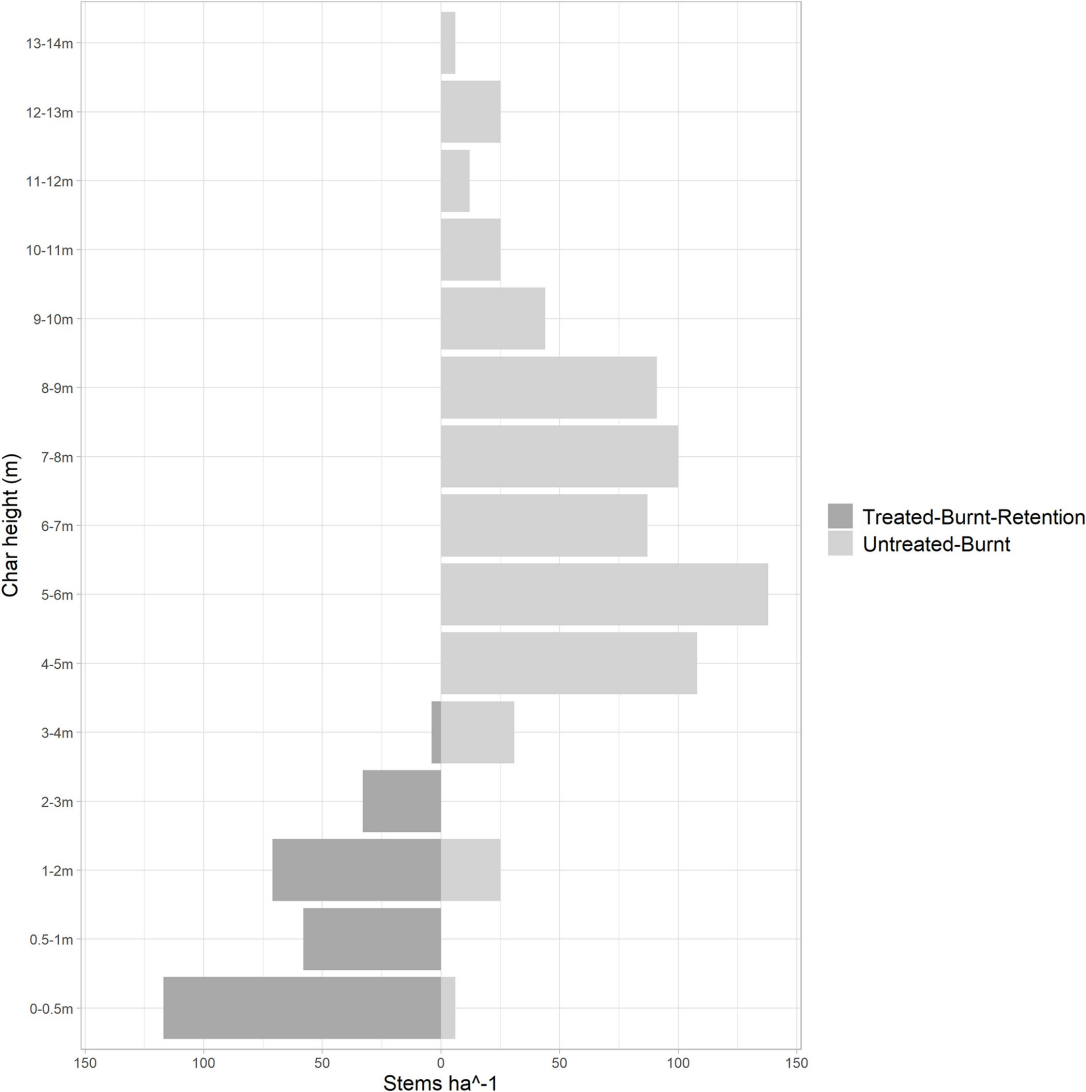
Total counts of char height in treated-burnt stand vs treated-unburnt stand by each height class.

The char height on the tree boles serves as a quantifiable indicator for assessing flame height in post-burn stands. It is evident that trees in the untreated-burnt stands had higher char heights, especially between 5 to 14m (Fig 6). More than 91% of the trees possessed bole char higher than 4 meters within the untreated-burnt stand, predominantly in the range of 4–9 meters, with 5–6 meters being the most frequent class (20%). Remarkably, some char heights exceeded 10m into the crown in the untreated-burnt stand.

In contrast, most trees in treated-burnt-retention stand possessed fire char at 0–0.5m (41%), followed by 1-2m (25%) and 0.5-1m (20%) (Fig 6). No fire scar higher than 4 meters was found in the treated-burnt-retention stand.

#### Fire behaviour along the direction of spreading

**Fig 7:**
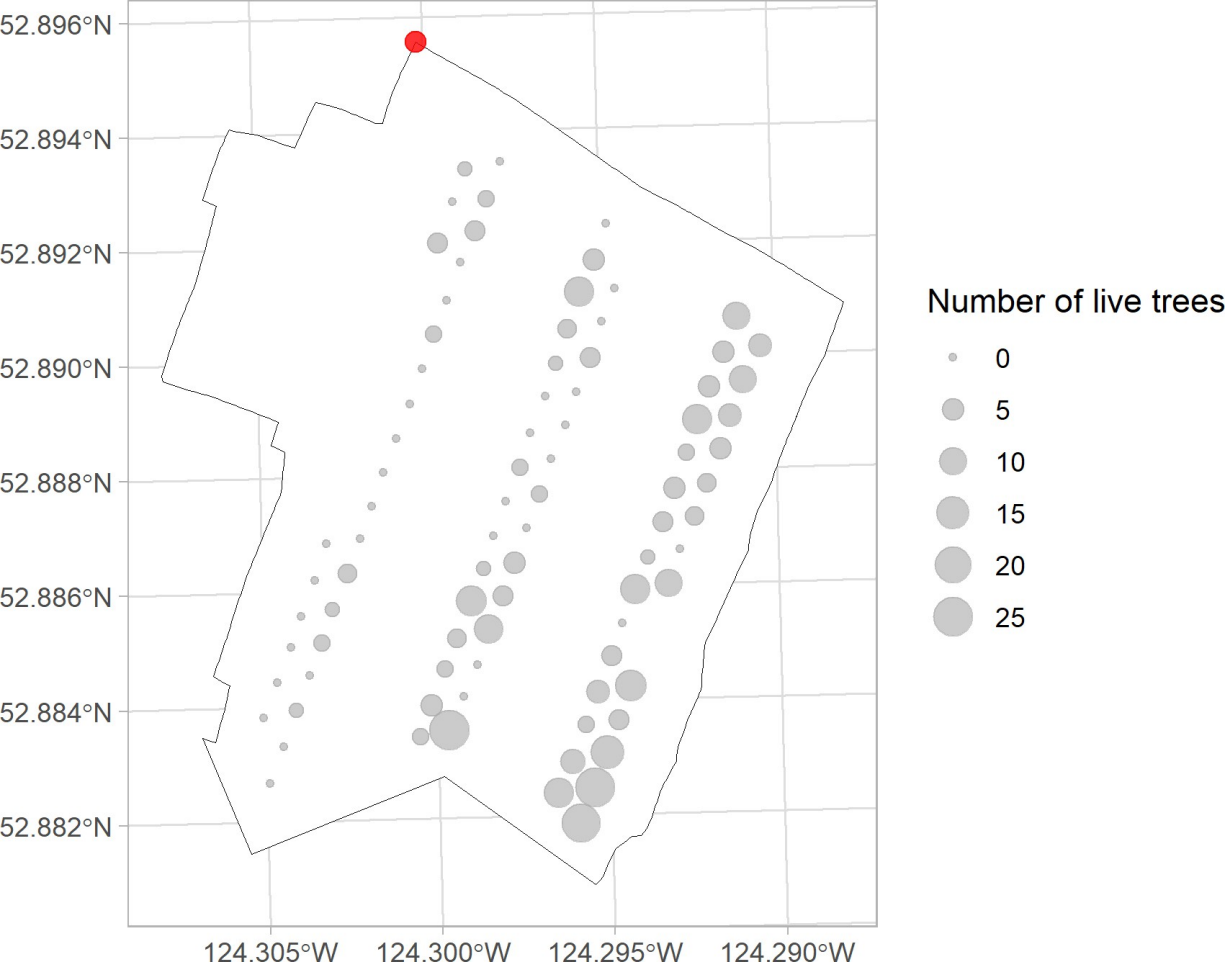
Bubble map showing the result of post-fire live tree tally in each retention patch along the three main skidding trails. Fire spread from the northwest corner to the southeast corner. Red dot on top left showing ignition spot.

**Fig 8:**
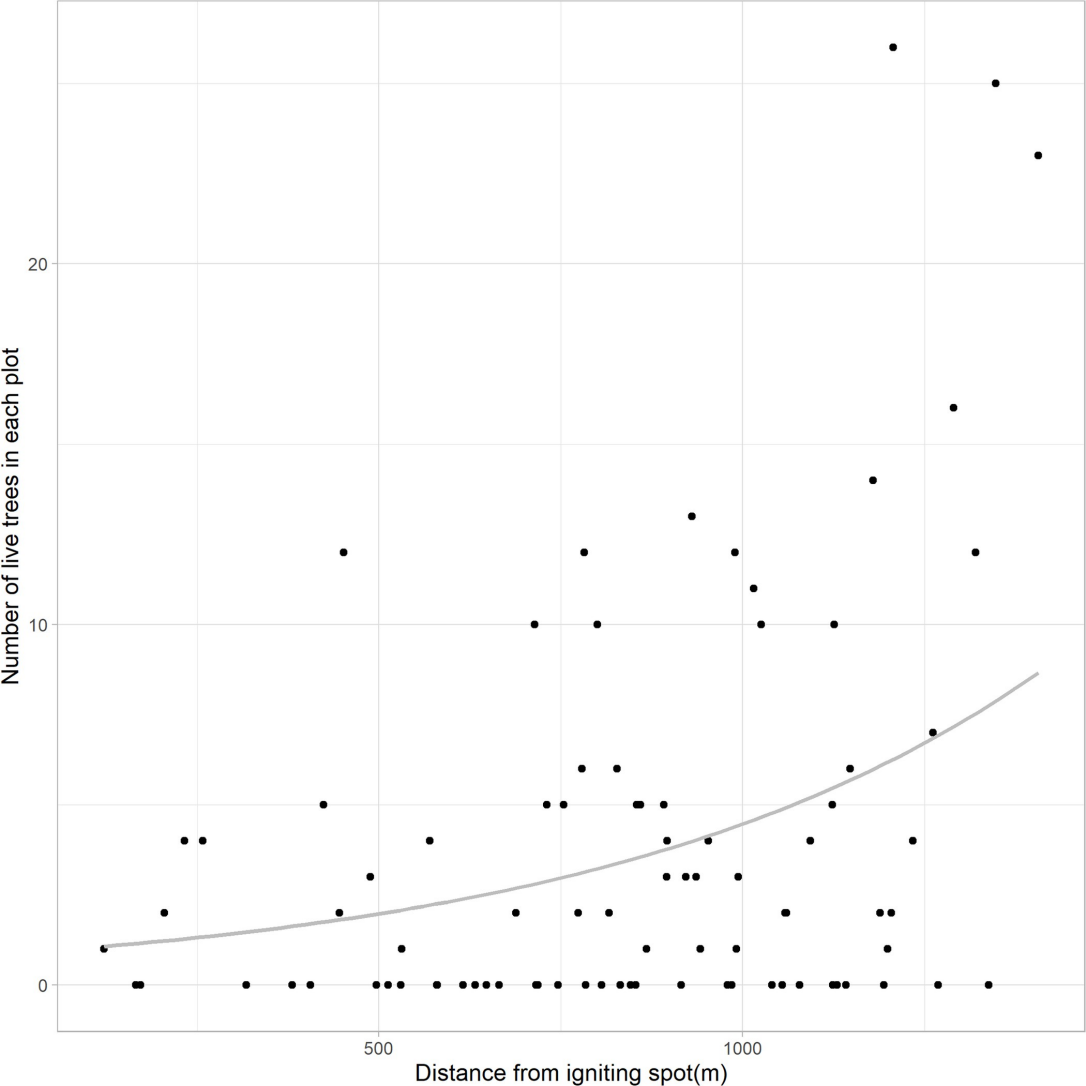
The distribution of ‘number of live trees’ over ‘distance from ignition’, with the fitted regression line using negative binomial model.

The results of the live tree tally are visualized using circles of different sizes that positively corelate to the number of live trees (Fig 7). The increasing circle sizes as fire travels further into the stand from the ignition spot indicated enhanced likelihood for trees to survive in the fire. No discernible pattern was found in the diagnostic plot that shows the deviance residuals over the fitted values. The deviance residuals are randomly scattered around zero. Homoscedasticity was satisfied given the constant spread of variance of the residuals across all levels of fitted values. The result suggested that the distance from ignition spot is significant in predicting the number of post-fire live trees in each patch (p = 0.0015). The fitted model was shown in the scatter plot with its slope being positive, indicating a positive correlation between the prediction and response variables (Fig 8).

**Fig 9:**
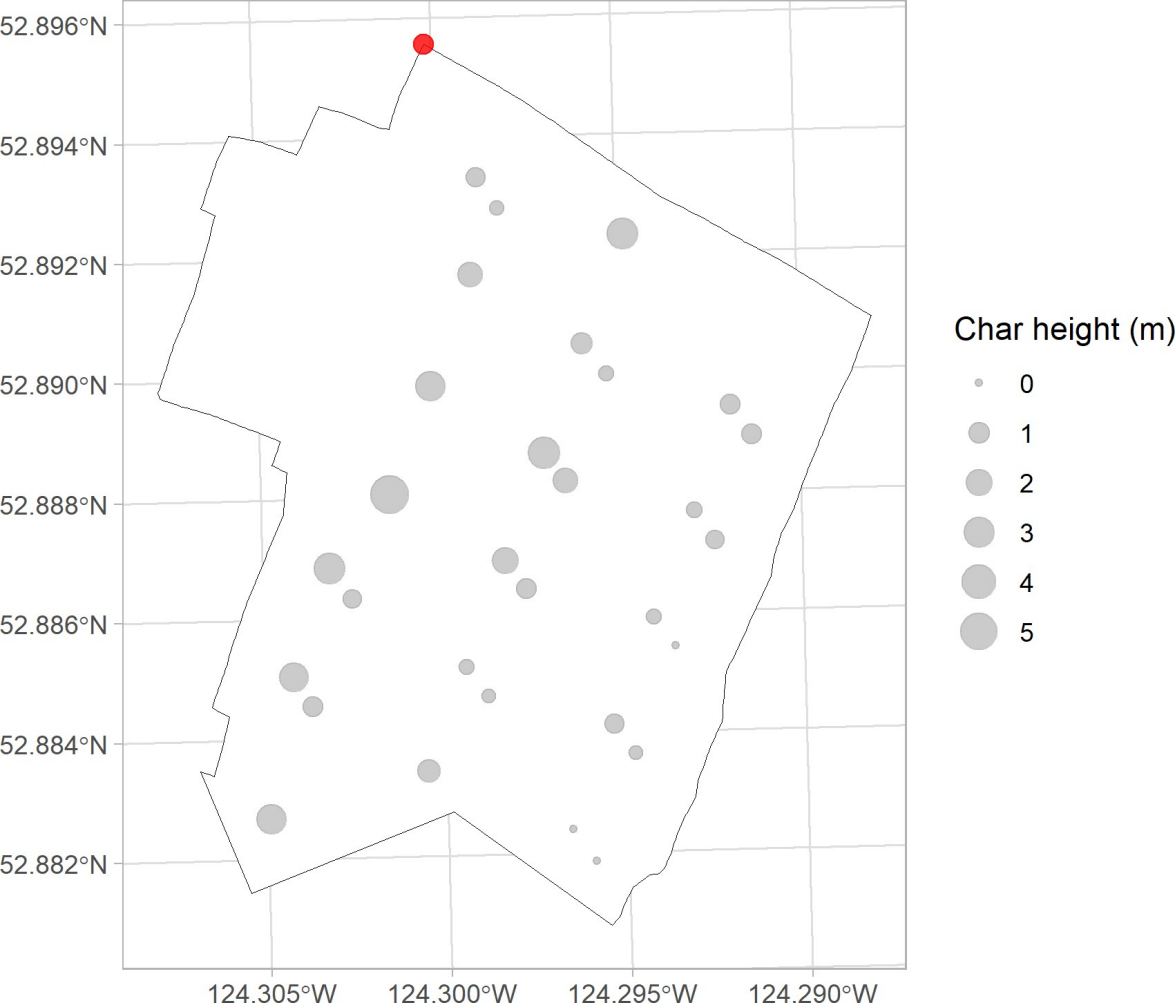
Bubble map showing the result of post-fire char height tally every 3rd retention patch along the three main skidding trails. Fire spread from the northwest corner to the southeast corner. Red dot on top left showing ignition spot.

The outcomes of the char height sampling along three skidding trails are presented in a similar way. The average char height from each plot tends to decrease as fire approaches further towards the southeast corner of the treated-burnt stand, shown by decreasing circle sizes (Fig 9). A negative linear correlation appears to exist between the distance and average char height in the scatter plot. However, this linear regression is not statistically significant (p > 0.05).

## Discussion

### Fire type difference in treated- and untreated-burnt stand

The distinction in bole char characteristics observed between treated and untreated burned stands suggests differing flame lengths and fire types across the two stands. In the treated-burnt stand, all post-fire trees exhibited bole char lower than 5 meters, which is indicative of a low- to moderate-intensity surface fire that primarily consumes fuels along the forest floor (Fig 6). In contrast, in the untreated-burnt stand, higher intensity fire is inferred to have occurred, given that the char heights exceeded 5 meters for nearly the entire stand. Remarkably, given the stand average height of 13-14m, the sparse presence of a few char heights greater than 12 meters suggests a very intense surface fire with some scattered passive and active crown fire across the untreated-burnt stand (Fig 6) [64]. Agee [65] concluded that the canopy structure is one of the most important factors that impact the fire behaviour. The results of our study are consistent with Agee’s findings, where we inferred differences in fire type between the treated- and untreated-burnt stands. We attribute the fire type difference to the fragmentation of stand crown resulting from the “checkerboard” treatment design of the irregular shelterwood. Within this design, the "openings" created by the irregular shelterwood system may have functioned as crown fuel mitigation patches, which disrupted the continuity of crown fuels. This break in continuity could result in the spreading fire moving back down to the surface, thereby moderating its behaviour as it moved through the openings. The finding demonstrated the potential of this irregular shelterwood system to reduce fire intensity. In line with this finding, reduced flame length and fire intensity was also recorded from treated stand in recent studies by Cram et al. [30] and Safford et al. [35]. This study provides more field evidence that fuel treatments aiming to alter canopy fuel structure can mitigate the impacts of fire, even under extreme conditions [34, 36]. It is recognized that this observed treatment effect may be temporally limited and may not apply uniformly across all ecosystems. Previous studies have reported increased surface wind speeds and elevated live and dead fuel loading in the open patches over longer time following harvest, due to the removal of overstory trees [84, 85]. These factors could potentially lead to more extreme fire behaviour, contrary to the reduced fire intensity observed in this study.

As this operational trial lacked controls, we acknowledge that the pre-fire fuel conditions, one critical side of the fire behaviour triangle [65], were not confirmed between treated- and untreated-burnt stand based on field measurements. Specifically, increased ground fuels, especially those below the 10000-hour class, are measured within treated-unburnt-retention patches compared to untreated-unburnt stand (Fig 5A–5D). This surface fuel accumulation is likely a result of the debris produced from harvesting in adjacent patches and the large downed woods resulting from both operational damage during harvesting the adjacent patches and windthrow at the edge of the retention patches, which has been documented in recent studies on post-treatment ground fuel dynamics in partial cuts [66–68, 86]. Additionally, untreated-unburnt stands displayed higher mean live tree density for smaller trees in layers 2 and 3, and higher mean pre-fire dead tree density compared to treated-unburnt-retention stands (Fig 4). Menning & Stephens [87] noted that the presence of small trees as ladder fuels would pose a greater potential to escalate fires to the canopy. However, their limited overall presence (<170 stem ha^-1^ with large SDs) led to minimal effect in altering intra-stand fire behaviour in our study. In summary, the existence of the two compounding factors–increased surface fuel loading and variable ladder fuels–cannot be overlooked. However, the difference in ladder fuels was not statistically significant. The increased amount of surface fuels is inevitably associated with this irregular shelterwood system given that no fuel treatment was conducted in the retention patches. Furthermore, while the surface fuel loading is higher in the retention patches, the changes to the continuity of canopy fuel likely overwrite this increased fire hazard, making the irregular shelterwood stand structure the most probable driver behind the fire type difference in treated- and untreated-burnt stands.

### Post-fire Survival in treated- and untreated-burnt stand

The treated-burnt stand displayed an increased number of live trees that withstood the fire in the retention patches compared to the untreated-burnt stand (Table 3). Although parallel studies on irregular shelterwood treated stands are limited, this finding agrees with recent research indicating decreased post-fire mortality in fuel treatment stands [31, 34–36]. However, the discrepancy in post-fire live trees between the two burnt stands (25 stems ha^-1^ in treated-burnt-retention vs 0 stem ha^-1^ in untreated-burnt) is less pronounced than observed in the aforementioned studies. This difference may be attributed to the excessive presence (around 50%) of beetle-induced snags in both treated- and untreated-burnt stands. It has been concluded by Jenkins et al. [88] that the beetle-attacked snags are highly combustible and contribute to extreme fire behaviour. The mortality difference between treated and untreated stands is expected to be more distinct should the number of pre-fire snags be reduced. Additionally, lodgepole pines inherently have very low fire tolerance due to their thin bark and dependency on fires to open their cones [89, 90]. Thus, expecting a substantial post-fire survival rate in lodgepole pine-dominated stands may be overly optimistic. In conclusion, the treated-burnt-retention stand displayed marginally lower post-fire tree mortality than the untreated-burnt stand, a difference expected to be more evident with fewer pre-fire snags on site. These results are specific to this local case and should not be generalized without further replicate studies.

### Distance effect in treated stand

The fitted negative binomial model suggested that the predictor variable—the distance from the plot center to the ignition spot–is significant in predicting the number of post-fire surviving trees within a plot (p < 0.01) (Fig 8), which is consistent with the field observations (Fig 7). The positive correlation between predictor and response reveals a diminishing fire severity as the fire spreads further into the irregular shelterwood treated stand, as evidenced by an increasing number of live trees in plots further away from the ignition point. In contrast, no discernible distance effect was observed in the untreated-burnt stand, given that the stand had 100 percent post fire mortality (Table 3). The relationship between post-fire survivors and distance in treated stand was also reported by Safford et al. [35] in their study on the impact of fuel treatment on fire spread, highlighting the role of stand structure in moderating fire behaviour. Comparable to the conditions in this study, complete canopy mortality was observed in untreated stand by Safford et al. [35], indicating the absence of distance effect. Similarly, a previous study [91] also found an increasing trend in post-fire tree survivorship in treated stand as fire traverses further in from the treatment boundary. This trend was even more distinct as the stand structural diversity increased [91]. The distance effect observed in this study and the literature further verified the positive effect of structural heterogeneity on regulating the impact of natural disturbances [92]. The skid trails are also believed to contribute to reducing and slowing the fire spread, strengthened by the fact there was mostly surface fire in the treated stand. The fire would have had to spot across each skid trail to spread. Due to the lack of real-time data, we acknowledge that the weather conditions could have jointly influenced the post-fire survivorship difference observed in the treated- and untreated-burnt stands. The differences in precipitation levels across the two stands could have contributed to varied survival rates at plots further from the ignition spot. However, this difference is expected to be minimal given the stands’ proximity to each other (3 km apart). The presence of extra smaller trees (layer 2 and 3) in the untreated-unburnt stand implies heavier ladder fuel loading in the pre-fire stage of untreated burnt stand [87]. Yet, as discussed above, the contribution of this factor is limited, considering the relatively insubstantial amount of ladder fuel. To summarize, the distance effect in this study inferred a positive structural effect in increasing tree survivorships in stands treated by an irregular shelterwood system. While this inference aligns with previous studies, a notable gap persists in the availability of comparative studies on direct treatment effect on wildfire behaviour across different treatment types.

In regard to the distance effect on char height, there appears to be a subtle decreasing trend in char heights as the fire progresses further into the stand (Fig 9). However, no statistical evidence was found to support the negative correlation between distance from ignition spot and average char height in the linear model (p = 0.099). Ritchie et al [91] noted a clearer decreasing trend in char height as fire spread into the treated stand, but likewise, the statistical relationships between the two variables were weak. There could be two potential reasons for this. Firstly, the structure of this stand may exert limited influence on reducing fire intensity, leading to the observed subtle changes in char heights. Secondly, the sample size for char heights measurement in the study may be too small to capture a sufficient number of observations needed to identify a significant underlying regression between char height and distance from ignition spot. Lastly, the process of bole char may present a random pattern influenced by the local accumulation of surface fuels at the trunk base and lichens along the tree trunk, resulting in variability in measured char height.

Due to the scarcity of comparable studies, it was not feasible to compare this finding with parallel investigations. Further studies will be required to understand the distance effect of this stand structure on fire intensity and to elucidate more conclusively on the treatment effect on fire behaviour.

### Management implications

Enhancing the resistance and resilience of forest ecosystems is a pivotal adaptive strategy to the adverse effects of climate change [93]. The results from this case study suggest the positive structural effect of an irregular shelterwood on promoting the resistance against wildfire, with resistance being defined as the capacity of a stand to persist during disturbance [94]. Our study found reduced char height in the irregular shelterwood retention patches, which reveals a reduced rate of spread and lower fire intensity. A positive relationship was also noted between the distance of the fire travelling into the stand and the number of post-fire live trees. This relationship is attributed to the presence of scattered small openings across the stand structure that were created approximately five years before the wildfire. While open patches in some ecosystems can increase fire line intensity due to fuel accumulation and wind exposure, in this study, the understory was given limited time to grow before the fire. Therefore, the creation of these openings reduced the fire’s rate of spread and limited its initial intensity as it approached the next adjacent retention patch. As fire spreads further into the irregular shelterwood stand, the cumulative effect of passing multiple openings eventually brings down the fire intensity to a level that allows some of the trees in each retention patch to survive. Consequently, stands of this structure are able to persist even in extreme fire seasons, fitting in the scope of forest resistance.

In the context of forest operation, this irregular shelterwood stand structure is achieved by a single-entry group harvesting of 0.15ha rectangular patches aiming for a 50% canopy opening intensity. The openings are 30m in width and 50m in length. Surrounding these openings are four retention patches of the same shape and size. This system does not plan a final cut for the retention patches, aiming to create structural diversity for 50–60 years following the single-entry. No additional treatment was implemented in the retention patches in this case, although removal of surface and ladder fuels can certainly further amplify the structural effect on reducing fire intensity. On a broader scope, this specific type of irregular shelterwood provides insights for wildfire operations that a 50% retention level achieved by multiple group openings on a single-entry harvest is effective in moderating the fire intensity during extreme seasons. It is acknowledged that this specific “checkerboard-pattern” irregular shelterwood system requires intense planning and operation, which may not be financially or operationally feasible for many occasions. However, in scenarios where fire risk management coincides with the preservation of mountain caribou habitat, particularly in flat, accessible terrain, this silviculture system may be the most suitable in meeting multiple management goals simultaneously. Such combination of terrain features and management objectives is often found in central interior BC in the Montane Spruce BEC zones where this irregular shelterwood system may be suitable to apply.

Broadly, implementing a range of silvicultural systems on the land-base can create more heterogenous forest conditions, especially in this study area landscape which is dominated by lodgepole pine, and provide the opportunity to evaluate the social acceptability of different systems. Specifically, the irregular shelterwood, as well of other partial harvesting methods, should be explored and operationally implemented based on this study to test their efficacy to adapt to climate change and escalating disturbances such as wildfires. This will be essential to balance the increasing need to manage forests for multiple objectives, such as wildlife habitat and timber, and ensuring resilience over the long-term.

### Limitation and future research

The study was executed to the highest standards within the constraints of available resources, adhering to scientific research design principles. However, it is acknowledged that improvements could be made to several aspects of this study to draw more general and causal inferences. First, the study was observational and was conducted over only one site with no replicates nor established controls for research experiment purpose. This means that the conclusions and inferences on all three discussed findings–fire type difference, post-fire survivorship in treated and untreated stands, and distance effect in treated stand–are confined to this specific site only and cannot be generalized.

It would have been the authors’ greatest interest to test the statistical significance of the treatment effect on predicting post-fire char height and survivorship. However, without replicates, any statistical test on treatment effects is unjustifiable. Consequently, all conclusions on treatment effect are inferred from post-fire evidence alone. Should more replicates be identified across the landscape, this study could have performed more comprehensive statistical tests on the treatment effects of this irregular shelterwood system and broaden the scope of the inferences to the landscape level.

Although the unpredictability of wildfires makes identifying replicates largely chance-dependent, incorporating more replicates through controlled, prescribed burns could address this limitation, especially given the established methodology of implementing the irregular shelterwood system in the Quesnel TSA. This paper outlines a methodology to assess the interaction between irregular shelterwood stand structure and fire dynamics, implying a potential increased resistance to wildfire in treated stand. Future research should focus on establishing causal links between treatment effects and fire dynamics through implemented burning trials at broader scales.

## Conclusion

In this study, we used a retrospective approach to assess the response and effects of fire on an untreated natural stand and an irregular shelterwood stand. The irregular shelterwood stand, which is still in its early stage with clean openings not yet occupied by regeneration, demonstrated its effectiveness in reducing wildfire intensity as assessed by char height compared to an untreated stand. We also observed decreasing char height from trees as wildfire travelled further into the stand. Although no statistical evidence was found to support that the irregular shelterwood system increases post-fire tree survivorship in this lodgepole pine stand. This case study provides field evidence that an irregular shelterwood can be an effective silvicultural system to promote wildfire resistance and resilience in lodgepole pine forests in interior BC before the openings are colonized by regeneration. Future studies are appealed to test the efficacy of the irregular shelterwood system against wildfire with more replicates at a broader scale across the landscape for causal inferences.
